# The Photosynthetic Characteristics of Leaves in Different-Colored *Brassica juncea*

**DOI:** 10.3390/plants14203197

**Published:** 2025-10-17

**Authors:** Gang Yang, Jiquan Zhang, Abbas Muhammad Fahim, Yuanyuan Zhang, Wancang Sun, Li Ma, Yuanyuan Pu, Lijun Liu, Wangtian Wang, Tingting Fan, Junyan Wu

**Affiliations:** 1 State Key Laboratory of Arid Land Crop Science, Gansu Agricultural University, Lanzhou 730070, China; yangg@gsau.edu.cn (G.Y.); 13830277694@163.com (J.Z.); fahimabbaskhan@yahoo.com (A.M.F.); 18293121851@163.com (W.S.); puyy@gsau.edu.cn (Y.P.); 2 College of Agronomy, Gansu Agricultural University, Lanzhou 730070, China; 17352234587@163.com (Y.Z.); mal@gsau.edu.cn (L.M.); liulj@gsau.edu.cn (L.L.); wangw@gsau.edu.cn (W.W.); 3 College of Life Science and Technology, Gansu Agricultural University, Lanzhou 730070, China

**Keywords:** *Brassica juncea*, leaf color, photosynthetic characteristics

## Abstract

Leaf color is a key trait influencing photosynthetic efficiency in plants. This study investigates the photosynthetic characteristics of differently colored leaves in *Brassica juncea* L. using green-leaved (SWJ) and purple-red-leaved (RLJ) varieties, their reciprocal F_1_ hybrids, and F_2_ populations. The results show that the net photosynthetic rate and chlorophyll content of SWJ were significantly higher than those of RLJ, while F_1_ hybrids exhibited intermediate photosynthetic performance. All five measured photosynthetic traits—net photosynthetic rate, stomatal conductance, intercellular CO_2_ concentration, transpiration rate, and chlorophyll content—segregated significantly in the F_2_ generation and were identified as quantitative traits. Notably, transpiration rate was positively correlated with leaf color, whereas no correlation was found with net photosynthetic rate or intercellular CO_2_ concentration. A key finding is the occurrence of purple-leaved plants with high photosynthetic rates and green-leaved plants with low photosynthetic rates in the F_2_ generation, indicating the potential to combine high photosynthesis with anthocyanin-rich purple leaves. This study provides new genetic insights and a theoretical basis for breeding high-yield, stress-tolerant *Brassica juncea* varieties.

## 1. Introduction

*Brassica juncea* (B. juncea, AABB, 2n = 36), a plant belonging to the genus Brassica of the family Brassicaceae, together with *Brassica napus* (*B. napus*, AACC, 2n = 38) and *Brassica rapa* (*B. rapa*, AA, 2n = 20) are the three major oilseed crops in the genus Brassica [1]. Besides being used for oil production, *Brassica juncea* is also an essential vegetable in China and can be used as a seasoning. In addition, *Brassica juncea* has excellent characteristics such as drought resistance, tolerance to barren soil, and resistance to diseases and pests, making it quite suitable for planting in mountainous areas [2].

Leaves are important vegetative organs for photosynthesis in plants, but there are great differences in morphology and color among different plant leaves [3]. The different colors of the leaves are mainly determined by the pigments in the leaves. The pigments in the leaves of higher plants are mainly divided into three categories: one is chlorophyll, which is a type of green pigment necessary for photosynthesis of higher plants and is mainly composed of chlorophyll a and chlorophyll b; the second is carotenoid, which is mainly composed of carotene and lutein; the third is flavonoid pigment [4]. The proportion of pigments in leaves of different colors is different, which will affect their photosynthetic efficiency. Studies have shown that high-yielding varieties are closely related to chlorophyll and photosynthetic efficiency [5,6]. When higher plants are photosynthesizing, light energy also affects the change in leaf color, and strong light can induce the accumulation of anthocyanins, which can make the leaf color of plants purple-red [7].

Purple-red-leaf *B. juncea* is a unique variety of mustard, and its leaves are pale red to purplish red throughout its growth and development. Studies have shown that the main components of red pigment are delphinin and cyanidin, both of which are anthocyanins [8]. Anthocyanins are one of the secondary metabolites of flavonoids, which play an important role in plant responses to abiotic stress [9]. Therefore, it is of great significance to study the traits and regulation mechanism of plant leaf color to improve plant light and efficiency and cultivate varieties with high light efficiency.

While leaf color variation is known to influence light absorption and stress tolerance, its genetic and physiological links to photosynthetic efficiency in *Brassica juncea* remain underexplored. This study uses reciprocal crosses between green (SWJ) and purple-red (RLJ) B. juncea to analyze the inheritance of photosynthetic traits and their correlation with leaf color, aiming to support breeding for high-efficiency, resilient varieties.

## 2. Results

### 2.1. The Characteristics of Brassica juncea Leaves with Different Colors

**Figure 1:**
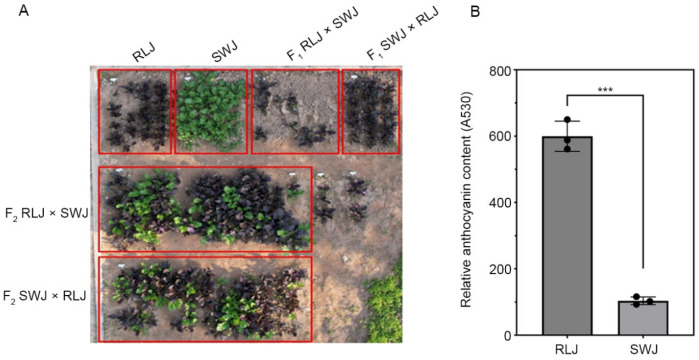
The leaf color phenotypes of the parental lines and the anthocyanin content. (**A**) Field phenotypic observations of parental plants, F_1_ generation plants, and F_2_ generation plants. (**B**) The anthocyanin content of the parental lines RLJ and SWJ (*** *p* < 0.001, non-parametric test).

The two parental lines used in this study, RLJ (purple-red leaves) and SWJ (green leaves), exhibited distinct leaf coloration (Figure 1A). Leaf coloration is closely associated with the pigment content and types in plant leaves. To investigate the differences in pigment content between RLJ and SWJ, we measured the relative anthocyanin content in their leaves. The results showed that the anthocyanin content in RLJ leaves was significantly higher than that in SWJ (Figure 1B). Both reciprocal F_1_ hybrids (RLJ × SWJ and SWJ × RLJ) displayed uniform purple leaf coloration (Figure 1A). When the two F_1_ hybrids were self-pollinated, the resulting F_2_ populations showed the segregation of leaf color phenotypes (Figure 1A).

### 2.2. Analysis of Photosynthetic Rate of Brassica juncea Leaves

**Figure 2:**
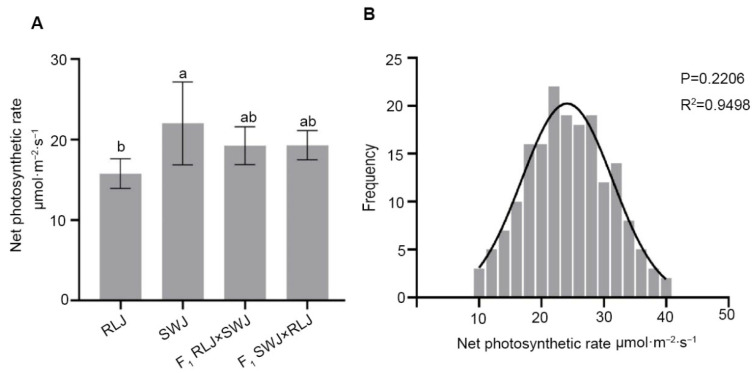
Comparison of the net photosynthetic rates of the leaves of the parental generation, the F_1_ generation from reciprocal crosses, and the F_2_ generation materials. (**A**) Statistical results of the net photosynthetic rate of the leaves of the parental generation and the F_1_ generation from reciprocal crosses. Significant differences (*p* < 0.05) are indicated by different lowercase letters. (**B**) Frequency distribution plots of net photosynthetic rates of F_2_ generation leaves.

The photosynthetic rate is an important indicator of photosynthetic efficiency in plants, reflecting their capacity to fix CO_2_ and synthesize organic compounds. In order to study the photosynthetic characteristics of leaves of *Brassica juncea* with different colors, the photosynthetic data of leaves of RLJ and SWJ, F_1_ and F_2_ generations were determined. As shown in Figure 2, the results showed that the net photosynthetic rate of the parent RLJ was between 18.63 and 13.99 μmol·m^−2^·s^−1^, while that of the parent SWJ was between 30.79 and 18.44 μmol·m^−2^·s^−1^, which was significantly higher than that of the RLJ. In the F_1_ generation, the net photosynthetic rate of RLJ × SWJ was between 21.92 and 15.82 μmol·m^−2^·s^−1^, and that of SWJ × RLJ was between 22.01 and 17.37 μmol·m^−2^·s^−1^.

The net photosynthetic rate of each leaf in the F_2_ generation population was significantly segregated, the value ranged from 9 to 40 μmol·m^−2^·s^−1^, and there were 3 plants close to 40 μmol·m^−2^·s^−1^. The number distribution analysis of the net photosynthetic rate of F_2_ generation showed that the normality detection *p* = 0.2206 > 0.05 conformed to the normal distribution. The normal distribution and high R^2^ value (0.9498) of net photosynthetic rate in the F_2_ population indicate that this trait is quantitatively inherited, which is likely controlled by multiple genes.

### 2.3. Stomatal Conductance Analysis of Brassica juncea Leaves

**Figure 3:**
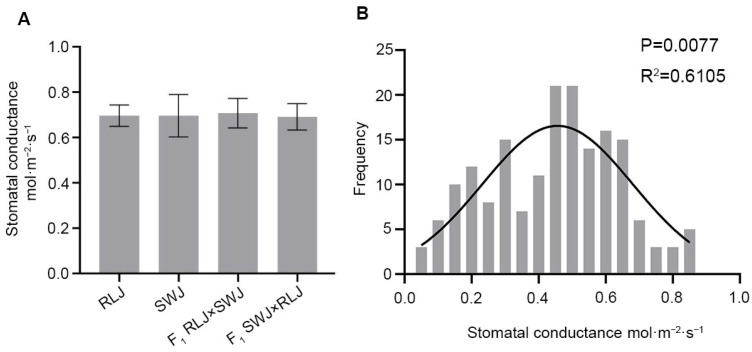
Comparison of the stomatal conductance leaves of the parental generation, the F_1_ generation from reciprocal crosses, and the F_2_ generation materials. (**A**) Statistical results of the stomatal conductance of the leaves of the parental generation and the F1 generation from reciprocal crosses. (**B**) Frequency distribution diagram of the stomatal conductance of the F_2_ generation material blades.

Stomatal conductance is an important parameter affecting the photosynthetic characteristics of leaves. It reflects the diffusion rate of gases (such as carbon dioxide and water vapor) through the stomata of leaves, and directly affects the supply of carbon dioxide inside the leaves and the loss of water. Stomatal conductance was analyzed in RLJ, SWJ and their reciprocal F_1_ hybrids. As shown in Figure 3, the stomatal conductance values ranged from 0.62 to 0.72 mol·m^−2^·s^−1^ for RLJ and from 0.62 to 0.85 mol·m^−2^·s^−1^ for SWJ. Variance and significance analysis revealed no statistically significant difference between the two parental lines. In the F_1_ hybrids, the stomatal conductance of RLJ × SWJ ranged from 0.613 to 0.79 mol·m^−2^·s^−1^, while that of the reciprocal cross SWJ × RLJ ranged from 0.62 to 0.78 mol·m^−2^·s^−1^. Comparative analysis indicated no statistically significant differences in stomatal conductance between the two F_1_ hybrids and their parental lines. The stomatal conductance analysis of 251 leaves in the F_2_ population showed that the stomatal conductance was significantly segregated, with values ranging from 0.045 to 0.866 mol·m^−2^·s^−1^, and 7 plants exceeded 0.8 mol·m^−2^·s^−1^. The normal distribution analysis of the stomatal conductance of the F_2_ generation showed that the *p* = 0.0077 > 0.05 and R^2^ = 0.6105, suggesting that stomatal conductance is quantitatively inherited, which is likely controlled by multiple genes.

### 2.4. Analysis of Intercellular CO_2_ Concentration in Brassica juncea Leaves

**Figure 4:**
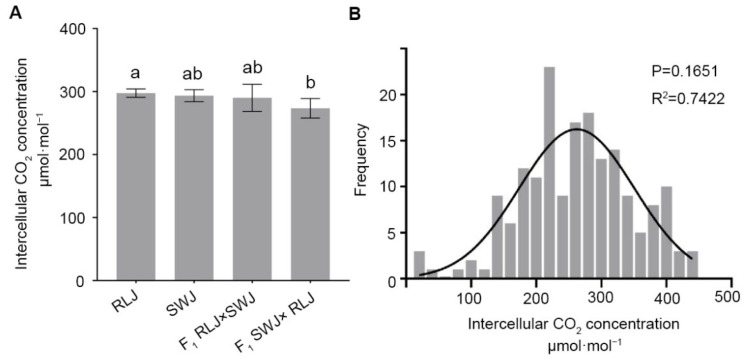
Comparison of the intercellular CO_2_ concentration of the leaves of the parental generation, the F1 generation from reciprocal crosses, and the F_2_ generation materials. (**A**) Statistical results of the intercellular CO_2_ concentration of the leaves of the parental generation and the F_1_ generation from reciprocal crosses. Significant differences (*p* < 0.05) are indicated by different lowercase letters. (**B**) Frequency distribution plots of intercellular CO_2_ concentration of F_2_ generation leaves.

The intercellular CO_2_ concentration (Ci) is a key parameter in the study of photosynthesis. It reflects the availability of CO_2_ inside the leaves and directly affects the photosynthetic rate. Intercellular CO_2_ concentration (Ci) was analyzed in RLJ, SWJ, and their reciprocal F1 hybrids. As shown in Figure 4, the Ci values of RLJ ranged from 409.90 to 433.60 μmol·mol^−1^, while those of SWJ ranged from 395.34 to 433.12 μmol·mol^−1^. Variance analysis and significance testing indicated no statistically significant difference in Ci between the two parental lines. In the F1 hybrids, the intercellular CO_2_ concentration (Ci) of RLJ × SWJ ranged from 374.32 to 437.28 μmol·mol^−1^, while that of the reciprocal cross (SWJ × RLJ) ranged from 370.68 to 420.11 μmol·mol^−1^. The results demonstrated that the Ci values of both F_1_ hybrids were consistently lower than those of either parent. Variance and significance analysis revealed an extremely significant difference in Ci between the F_1_ hybrid SWJ × RLJ and its paternal parent RLJ.

The analysis of the intercellular CO_2_ concentrations of 251 leaves in the F_2_ generation population showed that the intercellular CO_2_ concentrations were significantly segregated, ranging from 13.24 to 445.18 μmol·mol^−1^, and 6 plants were close to 445 μmol·mol^−1^. The normal distribution analysis of the intercellular CO_2_ concentration of the F_2_ generation showed that the *p* = 16516 > 0.05 and R^2^ = 0.7422, suggesting that the intercellular CO_2_ concentration is quantitatively inherited, likely controlled by multiple genes.

### 2.5. Analysis of Leaf Transpiration Rate of Brassica juncea

**Figure 5:**
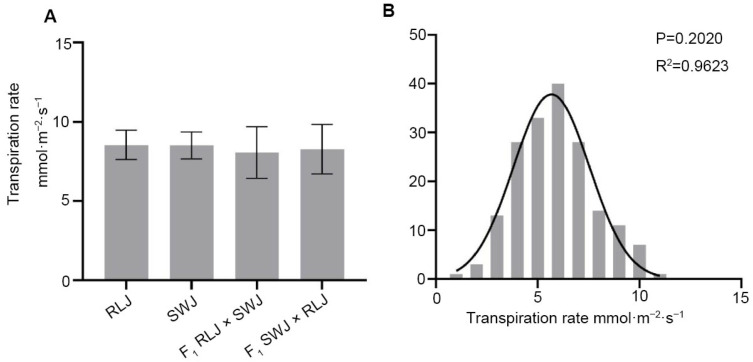
Comparison of the transpiration rate of the leaves of the parental generation, the F_1_ generation from reciprocal crosses, and the F_2_ generation materials. (**A**) Statistical results of the transpiration rate of the leaves of the parental generation and the F_1_ generation from reciprocal crosses. (**B**) Frequency distribution plots of transpiration rate of F_2_ generation leaves.

Transpiration is closely related to photosynthesis and is an important part of the water use efficiency of plants. As shown in Figure 5, the transpiration rate of RLJ and SWJ was analyzed, along with their reciprocal F_1_ hybrids. The transpiration rate values of RLJ ranged from 7.01 to 9.46 mol·m^−2^·s^−1^, while those of SWJ ranged from 7.15 to 9.37 mol·m^−2^·s^−1^. Variance analysis and significance testing indicated no statistically significant difference in transpiration rate between the two parental lines. In the F1 hybrids, the transpiration rate of RLJ × SWJ ranged from 5.74 to 10.40 mol·m^−2^·s^−1^, and that of the reciprocal cross (SWJ × RLJ) ranged from 5.65 to 9.43 mol·m^−2^·s^−1^. Comparative analysis revealed no significant differences in transpiration rate between either parent or their F_1_ hybrids.

The transpiration rate of 251 leaves per plant in the F_2_ generation population showed that the transpiration rate was significantly segregated, with values ranging from 1.28 to 10.85 mol·m^−2^·s^−1^, and 14 individual plants approaching 10 mol·m^−2^·s^−1^. The normal distribution analysis of the transpiration rate of the F_2_ generation showed that the *p* = 0.2020 > 0.05 and R^2^ = 0.9623, suggesting that the transpiration rate is quantitatively inherited, likely controlled by multiple genes.

### 2.6. Comparison of Chlorophyll Content in Brassica juncea Leaves

**Figure 6:**
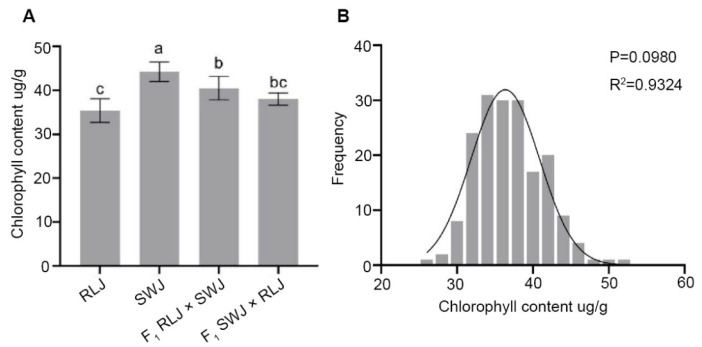
Comparison of the chlorophyll content of the leaves of the parental generation, the F_1_ generation from reciprocal crosses, and the F_2_ generation materials. (**A**) Statistical results of the chlorophyll content of the leaves of the parental generation and the F_1_ generation from reciprocal crosses. Significant differences (*p* < 0.05) are indicated by different lowercase letters. (**B**) Frequency distribution plots of chlorophyll content of F_2_ generation leaves.

Chlorophyll mainly exists in the chloroplasts of plants. It can absorb light energy, convert light energy into chemical energy, and drive the process of photosynthesis. Therefore, the chlorophyll content is often used as an important indicator to evaluate the photosynthetic capacity and physiological state of plants. As shown in Figure 6, the numerical analysis of chlorophyll content in the leaves of RLJ, SWJ, and their reciprocal crosses of F_1_ generation plants revealed that the chlorophyll content of RLJ ranged between 33.47 and 39.00 µg·g^−1^, while that of SWJ ranged between 41.90 and 47.53 µg·g^−1^. Variance and significance analyses demonstrated that SWJ exhibited significantly higher chlorophyll content compared to RLJ, with the difference being statistically extremely significant (*p* < 0.01). In the F_1_ generation, the chlorophyll content of the hybrid “RLJ × SWJ” ranged between 37.90 and 44.67 µg·g^−1^, while that of the reciprocal hybrid “SWJ × RLJ “ranged between 36.5000 and 40.1667 µg·g^−1^. The results indicate that the chlorophyll content of both F_1_ hybrids fell between the values of the two parental lines. Variance and significance analyses demonstrated that the chlorophyll content of the F_1_ generation plants was significantly lower than that of SWJ, with an extremely statistically significant difference (*p* < 0.01). Additionally, the chlorophyll content of the hybrid “RLJ × SWJ” was significantly higher than that of the RLJ parent, also exhibiting an extremely statistically significant difference (*p* < 0.01).

Analysis of chlorophyll content in the leaves of 251 individual plants from the F_2_ generation revealed significant segregation, with values ranging from 26.47 to 51.37 µg·g^−1^. Notably, 8 individual plants exhibited chlorophyll content close to 51 µg·g^−1^. The normal distribution analysis of chlorophyll content of the F_2_ generation showed that the *p* = 0.0980 > 0.05 and R^2^ = 0.9324, suggesting that chlorophyll content is also quantitatively inherited, likely controlled by multiple genes.

### 2.7. Correlation Analysis of Photosynthetic Characteristics of Brassica juncea with Different Leaf Colors

**Figure 7:**
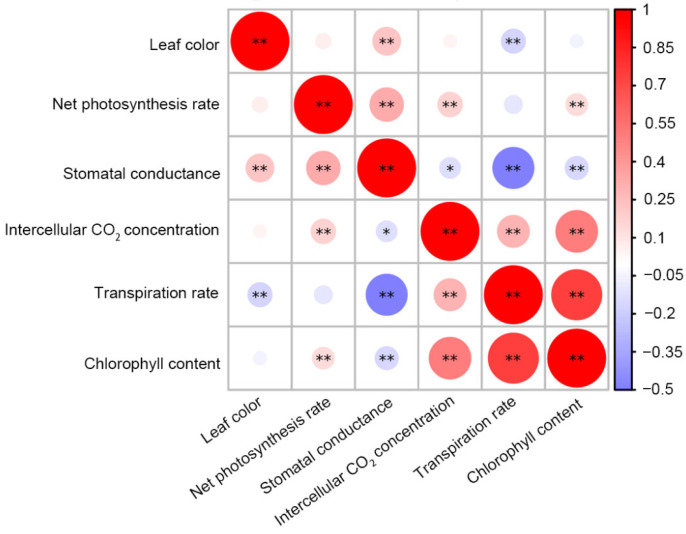
Correlation analysis of photosynthetic characteristics of *Brassica juncea* with different leaf colors (* *p* < 0.05 and ** *p* < 0.01).

Leaf color was coded as a binary trait (0 for green, 1 for purple) for correlation analysis. Leaf color was positively correlated with stomatal conductance and negatively correlated with transpiration rate, suggesting that purple leaves may have distinct stomatal behavior (Figure 7). The net photosynthetic rate was positively correlated with stomatal conductance, intercellular CO_2_ concentration, and chlorophyll content. Interestingly, a negative correlation was observed between stomatal conductance and transpiration rate, suggesting that transpiration rate is not only controlled by stomatal conductance but also strongly depends on meteorological drivers, primarily vapor pressure deficit and air movement. Therefore, this result may be related to the climatic conditions during the measurement of the photosynthesis data.

## 3. Discussion

In this study, it was found that there was a great difference in the photosynthetic characteristics between SWJ and RLJ. Among them, the net photosynthetic rate and chlorophyll content of SWJ were significantly higher than those of RLJ, which may be the main reason why the photosynthetic characteristics of SWJ were better than those of RLJ. The net photosynthetic rate of the F_1_ generation is between that of the two parents, reflecting the intermediate characteristics of the photosynthetic performance of the hybrid offspring. The net photosynthetic rate of the F_2_ generation shows obvious segregation and conforms to a normal distribution, indicating that it is a quantitative trait. This means that it is jointly regulated by multiple genes and environmental factors, providing a rich genetic variation basis for improving the photosynthetic rate through crossbreeding [10].

There were no significant differences in stomatal conductance, intercellular CO_2_ concentration, and transpiration rate. However, all these traits in the F_2_ generation exhibited quantitative characteristics and segregation occurred. The segregation of photosynthetic traits in the F_2_ population underscores their polygenic nature. Stomatal conductance affects the rate at which CO_2_ enters the leaves. The segregation of this trait in the F_2_ generation indicates that there is rich genetic diversity for stomatal conductance. Through selective breeding, the stomatal function can be optimized, which in turn affects the photosynthetic efficiency [11]. The intercellular CO_2_ concentration is an indicator of the concentration of raw materials for photosynthetic carbon assimilation. Strangely, we found that some individual plants in the F_2_ generation had intercellular CO_2_ concentrations exceeding 400 μmol·mol^−1^, which may be related to respiratory CO_2_ release or minor environmental fluctuations during measurements. The significant segregation of this trait in the F_2_ generation and its correlations with other photosynthetic parameters imply that the photosynthetic performance can be enhanced by regulating this indicator. The transpiration rate not only participates in water metabolism but is also related to heat dissipation and gas exchange during the photosynthetic process. Its characteristics as a quantitative trait and the segregation observed in the F_2_ generation provide the possibility for breeding *Brassica juncea* varieties that are adapted to different environments [12].

There is a significant positive correlation between leaf color and transpiration rate. This result reveals the inherent connection between leaf color and the photosynthetic physiological process. Purple leaves may affect these photosynthesis-related indicators through a certain mechanism, or they may be influenced by common genetic or physiological regulatory pathways. The significant negative correlation between transpiration rate and stomatal conductance is different from what was expected. It is likely that there are other factors limiting the transpiration rate in leaves, such as the meteorological drivers, primarily vapor pressure deficit, and air movement. Further research is needed to analyze these factors in more detail [13,14,15]. The positive correlation observed between chlorophyll content and net photosynthetic rate (Pn) is the classic and expected relationship in plant physiology. This can be explained by the fundamental role of chlorophyll: light harvesting, electron transport, and correlation with photosynthetic machinery. The negative correlation between chlorophyll and stomatal conductance suggests that photosynthesis is a multi-step process that can be limited by factors other than light capture. The correlations among photosynthetic traits and leaf color suggest pleiotropic or linked genetic control. The quantitative nature of these traits supports a polygenic inheritance model, where gene networks influence both pigment biosynthesis and photosynthetic efficiency.

Anthocyanins are water-soluble pigments present in plants, mainly accumulated in the vacuoles of plants, and have protective physiological functions in plants, such as preventing plant diseases and resisting the damage of adversity to plants [16,17]. Anthocyanins on the surface of plant tissues can reflect part of the light energy and reduce the damage of bright light to plant leaves [18,19]. Hughes found that anthocyanins in the leaf epidermis can shield some excess light energy, thereby playing a photoprotective role in the internal tissues of the leaf [20]. Chlorophyll is an important pigment involved in photosynthesis in plant chloroplasts, and its function is to capture light energy and drive the transfer of electrons to reaction centers. It has been shown that, to a certain extent, a decrease in chlorophyll content leads to a decrease in light energy absorption, which inhibits photosynthesis [21,22]. The individual plant statistics of F_2_ generation plants showed that the F_2_ generation plants showed trait separation, and purple-leaved high-photosynthetic plants and green-leaved low-photosynthetic plants appeared. Previous studies have found that the purple leaf traits in RLJ are formed by anthocyanins [23]. Anthocyanins are one of the products in the secondary metabolic pathway of flavonoids, which can reflect red, blue, purple, and red-violet colors. This is the main reason for the formation of leaf color and flower color [24,25]. In addition, anthocyanins have also been reported to be effective in scavenging leaf free radicals and playing an antioxidant protective role [26], indicating that anthocyanins in purple leaves likely offer photoprotective benefits, which could be leveraged in breeding programs targeting arid or high-light regions.

In this study, the F_1_ and F_2_ generation population obtained by the positive and negative crosses of the two parents was assessed, and only the population photosynthetic characteristics and individual plant trait statistics were determined. In the later stage, it is necessary to select a single plant for the F_2_ generation, screen out a single plant with high photosynthesis with purple leaves, and then carry out multiple inbreeding to breed excellent varieties with high photosynthesis and purple leaves that can be stably inherited. This will effectively expand the germplasm resources of *Brassica juncea*, and also provide an important theoretical basis for the breeding of stress resistance and high light efficiency in *Brassica juncea*.

## 4. Materials and Methods

### 4.1. Experimental Materials

The experimental materials used in this study primarily included purple-red-leaved RLJ, green-leaved SWJ, and the F_1_ and F_2_ populations constructed by crossing RLJ and SWJ as parental lines. Detailed information on the experimental materials is provided in Table 1. In this study, 133 individual plants were obtained from the F_2_ population of RLJ × SWJ, and 118 plants from the reciprocal F_2_ population of SWJ × RLJ. Due to the limited population size of each F_2_ group, the two populations were pooled (total 251 plants) for subsequent analysis of photosynthetic characteristics.

### 4.2. The Experimental Site

The experiment was conducted on 20 September 2023 at the on-campus experimental base of Gansu Agricultural University. The test area belongs to the middle temperate zone and is arid, with an average altitude of 2000 m, a pH of about 8.2, an average annual solar radiation of 141.6 kcal/cm^2^, a sunshine hour of 2476.6 h, an average annual temperature of 6.4 °C, an accumulated temperature of 2933.5 °C at ≥0 °C, and an accumulated temperature of 2239.1 °C at ≥10 °C. The frost-free period was 140 days. The average annual precipitation is 390.9 mm, the annual evaporation is 1531 m, the dryness is 2.53, the precipitation with 80% guaranteed rate is 365 mm, and the coefficient of variation is 24.3%. The soil is a typical loess soil, with soft soil, deep soil layer, uniform texture and good water storage performance.

### 4.3. Determination of Relative Anthocyanin Content

Regarding the determination of relative anthocyanin content using the single-wavelength method, the steps are as follows: weigh 0.2 g of fresh leaf tissue and grind it into fine powder using liquid nitrogen, and then add 5 mL of acidic ethanol solution (1% HCl in ethanol) and incubate it in the dark for 4 h to extract anthocyanins. Centrifuge the extract at 8000 rpm for 10 min and collect the supernatant, and measure the absorbance (A) of the supernatant directly at 530 nm using a spectrophotometer. Express the relative anthocyanin content as the absorbance value (A) obtained at the single wavelength (530 nm).

### 4.4. Determination of Photosynthetic Characteristics and Chlorophyll Content

After the 50th day of sowing, the photosynthesis intensity of fully expanded leaves of SWJ, RLJ, F_1_ and F_2_ generations was measured by a portable photosynthetic measurement system Li-6400 (LI-COR, Lincoln, NE, USA) from 10:00 to 12:00. LI-6400 Settings: PPFD = 1000 µmol·m^−2^·s^−1^, CO_2_ concentration = 400 µmol·mol^−1^, relative humidity = 50–60%, leaf temperature = 25 °C. The gas flow rate was controlled at 500 mmol/s, and the other parameters were set to the natural environment values. It was necessary to select the leaves in the plant that are fully spread out and in good growth condition, and measure each leaf six times to take the average value. The measured parameters included net photosynthetic rate, transpiration rate, intercellular CO_2_ concentration, and stomatal conductance, and were calculated and analyzed.

Chlorophyll Measurement: Chlorophyll content was measured using a portable chlorophyll meter (TYS-B, Zhejiang Top Yunong Technology Co., Ltd. of China), and values were converted to µg·g^−1^ based on a standard calibration curve from extracted samples.

Each genotype was measured with at least 3 biological replicates.

### 4.5. Statistical Analysis

Data were analyzed using SPSS 18.0. One-way ANOVA with LSD post hoc test was used to compare means among genotypes. Linear correlations between traits were assessed using Pearson’s correlation coefficient. Significance was set at *p* < 0.05.

## 5. Conclusions

This study demonstrates that photosynthetic traits in B. juncea are quantitatively inherited and exhibit significant genetic variation in segregating populations. The identification of high-photosynthetic purple-leaf individuals in the F_2_ generation provides a genetic resource for breeding programs aiming to combine high yield with stress tolerance. These findings underscore the potential of marker-assisted selection for improving photosynthetic efficiency in mustard crops.

## Figures and Tables

**Table 1:** The experimental material used in present study.

Material Name	Leaf Color	Origin
RLJ	Purple	Hunan Agricultural University
SWJ	Green	Shaanxi Academy of Agricultural Sciences
F_1_ RLJ × SWJ	Purple	Rapeseed Genetic Breeding Laboratory of Gansu Agricultural University
F_1_ SWJ × RLJ	Purple	Rapeseed Genetic Breeding Laboratory of Gansu Agricultural University
F_2_ RLJ × SWJ	Purple, Green	Rapeseed Genetic Breeding Laboratory of Gansu Agricultural University
F_2_ SWJ × RLJ	Purple, Green	Rapeseed Genetic Breeding Laboratory of Gansu Agricultural University

## Data Availability

All data generated or analyzed during this study are included in this published article.
